# Optimized alamarBlue assay protocol for drug dose-response determination of 3D tumor spheroids

**DOI:** 10.1016/j.mex.2018.07.011

**Published:** 2018-07-23

**Authors:** Christoph Eilenberger, Sebastian Rudi Adam Kratz, Mario Rothbauer, Eva-Kathrin Ehmoser, Peter Ertl, Seta Küpcü

**Affiliations:** a Institute of Applied Synthetic Chemistry and Institute of Chemical Technologies and Analytics, Faculty of Technical Chemistry, Vienna University of Technology, Vienna Getreidemarkt 9/163, 1060 Vienna, Austria; b Institute of Synthetic Bioarchitectures, Department of Nanobiotechnology, University of Natural Resources and Life Sciences, Vienna, Muthgasse 11, 1190 Vienna, Austria

**Keywords:** AlamarBlue proliferation assay, Cell culture, Tumor spheroids, AlamarBlue, Metabolic activity, Dose-response

## Abstract

The assessment of drug-dose responses is vital for the prediction of unwanted toxicological effects in modern medicine. Three-dimensional (3D) cell cultures techniques can provide *in vivo*-like spheroids and microtissues that resemble natural tumor function. However, formation of necrotic core and diffusion limitation of chemical compounds within these models can reduce the reproducibility and precision of standard bioassay protocols used to test two-dimensional (2D) cell cultures. Nonetheless, the accurate prediction of detrimental effects of test compounds based on functional bioassays is essential for the development of new efficient therapeutic strategies. For instance, alamarBlue^®^ is a widely-used commercially available redox indicator dye that can evaluate metabolic activity and cellular health status in a single-step procedure however, suitability and optimization of this bioassay must be determined for each individual application scenario. Here, we optimized the standard alamarBlue^®^ proliferation/viability protocol for tumor spheroid cultures to enhance assay precision during toxicological drug screening.

We optimized the original protocol of alamarBlue^®^ assay that usually suggests an incubation time of 2–4 hours. The key modifications of the protocol for spheroid cultures are as follows:

• Aspiration of cell culture medium before drug exposure.
• Replacement of drug-supplemented medium with 10% (v/v) alamarBlue^®^ reagent mixed with culture medium.
• Increase of incubation period to 24 h at 37 °C protected from light.

## Graphical abstract

**Figure:**
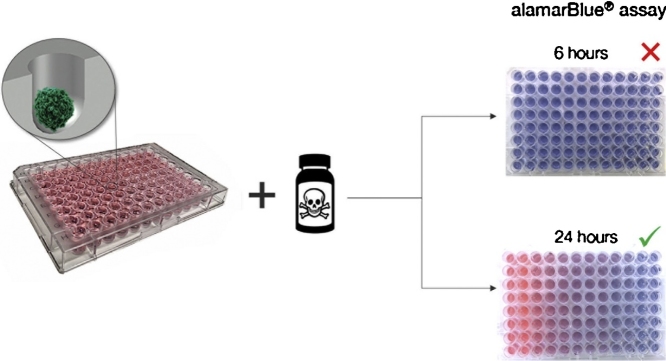

Specifications Table

**Table:** 

Subject area	• *Pharmacology, Toxicology and Pharmaceutical Science*
More specific subject area	*Cell biology, Tissue engineering*
Method name	*AlamarBlue proliferation assay*
Name and reference of original method	[[1], [2], [3]]
Resource availability	https://www.thermofisher.com/at/en/home/references/protocols/cell-and-tissue-analysis/cell-profilteration-assay-protocols/cell-viability-with-alamarblue.html

## Method details

### Preparation and generation of spheroid cultures

#### Materials

• Cell culture facility equipped with a CO_2_ incubator, laminar flow hood, bright-field microscope, a centrifuge and a cell counter.
• Plastic consumables: cell culture dishes and flasks, serological pipettes, syringes and centrifuge tubes.
• Hepatocellular carcinoma cells (HepG2).
• Cell culture medium: Minimal essential medium supplemented with 10% v/v of fetal bovine serum, 1%vol. of 20 mM L-glutamine and 1%vol. of 100 mM penicillin and streptomycin.
• Dulbecco’s Phosphate Buffered Saline (PBS) 1X (pH 7.1–7.4).
• Trypsin-EDTA solution (0.25%).
• Trypan blue stain 0.4%.

#### Procedure

Cells taken for experiments should be at log-phase of growth, approx. 60–80 % confluent. Amounts of media given for 75 cm^2^ cell culture flasks. All media applied to cells should be pre-warmed to 37 °C. (*Note*: Spheroid cultivation time, as well as morphology, can vary for different cell types. Therefore, initial cell seeding density should be pre-screened to identify the optimal cell density.)

1 Remove medium from cell culture. Wash the cells with PBS.
2 Detach cells using 5 mL trypsin solution. Incubate for at least 5 min at 37 °C until cells detach from the surface.
3 Add 5 mL of cell culture medium.
4 For trypsin removal transfer the suspension into 15 mL Falcon tube and centrifuge for 5 min at 294×*g*.
5 Gently remove supernatant and add 5 mL fresh medium.
6 Push the cell solution through a needle by using a syringe to dissociate larger aggregates into individual cells.
7 Mix 10 μL cell solution and 10 μL Trypan Blue in an Eppendorf tube and transfer 10 μL of the mix to a cell counter slide.
8 Measure cell number and viability and adjust the suspension to a cell density between 5.000 and 25.000 cells/mL.
9 For monolayer culture pipette 15.000 HepG2 cells/mL in each flat-bottom well of a tissue culture-treated 96-microwell plate.
10 For spheroid generation add 200 μL of cell suspension at a concentration of 15.000 cells/mL to each well of cell-repellant microwell plates. For our experiments, U-bottom 96-well plates were coated by a self-assembled anti-fouling nanobiointerface based on surface layer proteins as reported elsewhere [4,5].
11 Centrifuge the microwell plate for 10 min at 294 x g (*Note:* optional step; generates more uniformly shaped spherical spheroids for HepG2 cells).
12 Incubate cell cultures at 37 °C in 5% CO_2_ humidified atmosphere for 6 days. (*Note:* Experiments showed that medium exchange has no influence on cell viability of the spheroid. Therefore, fresh medium has not to be added).

### AlamarBlue^®^ protocol for drug dose-response evaluation

To assess dose-dependent toxic effects on HepG2 cells, the FDA-approved anti-liver cancer drug sorafenib was chosen. The compound inhibits tumor-cell proliferation and tumor angiogenesis and increases the rate of apoptosis [6]. (*Note*: Drug-dose response can vary for different cell types and drugs therefore, different responses must be pre-screened for 2D and 3D cultures.)

#### Materials

• Spectroscopic plate reader, CO_2_ incubator.
• Plastic consumables: cell culture microplates and flasks, serological pipettes, syringes.
• Cell culture media.
• Sorafenib Stock solution (1 mM) in DMSO.
• AlamarBlue^®^ reagent.

#### Procedure

1 Sorafenib was stored as 10 mM aliquots in DMSO at −20 °C and diluted to a working concentration in respective cell culture medium before drug exposure of HepG2 spheroids.
2 For determining the effect of a test agent on cell growth, ensure correct controls are included (e.g. untreated control, background fluorescence of phenol-red containing-medium, background fluorescence of alamarBlue®-containing medium).
3 After cultivation time of 6 days, remove cell culture medium gently by syringe and add diluted test compounds to wells and incubate cells. (*Note:* Remove liquids gently by using a syringe. Be aware to hold the needle in the opposite direction of the spheroid otherwise, it can be aspirated or destroyed; Cultivation time can vary based on cell concentration and type and should be initially checked for spheroid uniformity and shape).
4 In our experiments, cells were treated with different concentrations of sorafenib (0–1000 μmol/L) in triplicates for 24 h.
5 Incubate cell cultures at 37 °C and 5% CO_2_.
6 After incubation, aspirate compounds gently with a syringe to avoid interference with the proliferation assay due to physical cell damage.
7 Prepare fresh cell culture medium and add alamarBlue^®^ in an amount equal to 10% of the total volume. Mix the alamarBlue^®^ reagent by shaking.
8 Add 200 μL of alamarBlue^®^ -containing medium mix to each well.
9 Incubate spheroid cell cultures for 24 h at 37 °C protected from light.
10 Measure cytotoxicity/proliferation using fluorescence spectrophotometry and read fluorescence at excitation wavelength of 560 nm and emission wavelength of 590 nm.

To calculate percent difference in reduction between treated and control cells in cytotoxicity/ proliferation assays use the following formula:

$$\%.viability=.\frac{Experimental.RFU.with.chemical.compound}{Untreated.cell.control.RFU.value.}\times 100$$

### Qualitative evaluation of spheroid viability

#### Materials

• Inverted fluorescence microscope coupled with data analysis software.
• Syringe, needle, Eppendorf tubes.
• 4 mM Calcein acetoxymethyl (AM) in dimethyl sulfoxide (DMSO).
• 2 mM Ethidium- homodimer-1.
• PBS 1×.

#### Procedure

1 Mix 2 μL of 4 mM Calcein AM and 4 μL of 2 mM Ethidium homodimer-1 and fill up with PBS to a final volume of 1 mL.
2 After drug exposure, remove cell culture medium.
3 Wash spheroids with 200 μL PBS.
4 Remove the PBS gently by syringe.
5 Add 100 μL of the staining solution.
6 Incubate the cells for 30 min at 37 °C and protect from light.
7 Monitor live/dead cells by using a fluorescence microscope with respective fluorescence filter for Calcein AM (ex 485 nm, em 530 nm) and ethidium bromide EthD-1 (ex530 nm, em 645 nm).

## Method validation

**Fig. 1:**
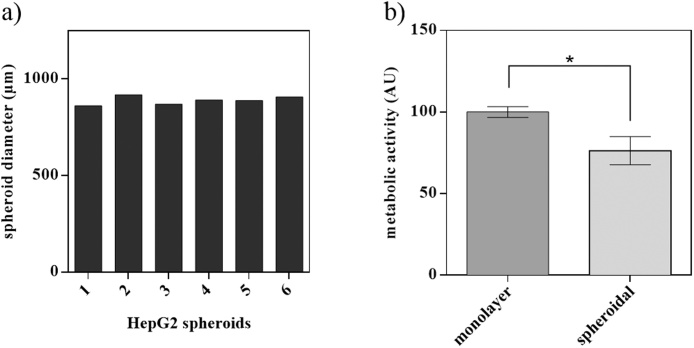
(a) Uniformity of six different HepG2 spheroid samples at an initial seeding density of 15.000 cells/mL at day 6 post-seeding. (b) Metabolic activity of monolayer compared to spheroidal cultures after 4 h incubation with alamarBlue^®^ at day 6 post-seeding (n = 3; *p < 0.05). Data points are expressed as mean values ± SD.

First, we tested spheroid uniformity as well as the response of the alamarBlue^®^ assay for 2D cell culture and 3D spheroids viability measurements. Uniform HepG2 spheroid size was observable after centrifugation with a spheroid diameter of 887.3 ± 30 μm. In addition, HepG2 spheroids exhibited a 24% decrease in metabolic activity compared to 2D cell culture after 4 h of incubation (Fig. 1). This effect stemming from spheroidal cultivation must be considered carefully for a comparative analysis of 2D and 3D cultures using alamarBlue^®^ assay.

**Fig. 2:**
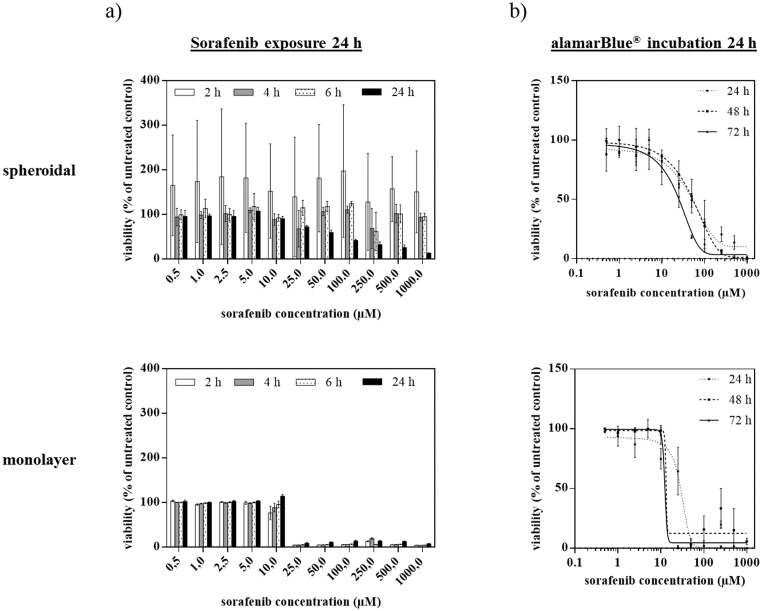
(a) Sorafenib dose-response after 24 h of exposure for 2–24 h alamarBlue^®^ incubation of HepG2 spheroids and monolayer cultures day 6 post-seeding (n = 3). (B) Dose-response of 24 h, 48 h and 72 h of HepG2 spheroid and monolayer cultures for sorafenib concentrations up to 1 mM using the optimized alamarBlue^®^ assay protocol after day 6 post-seeding (n = 3). Data points are expressed as mean values ± SD.

**Fig. 3:**
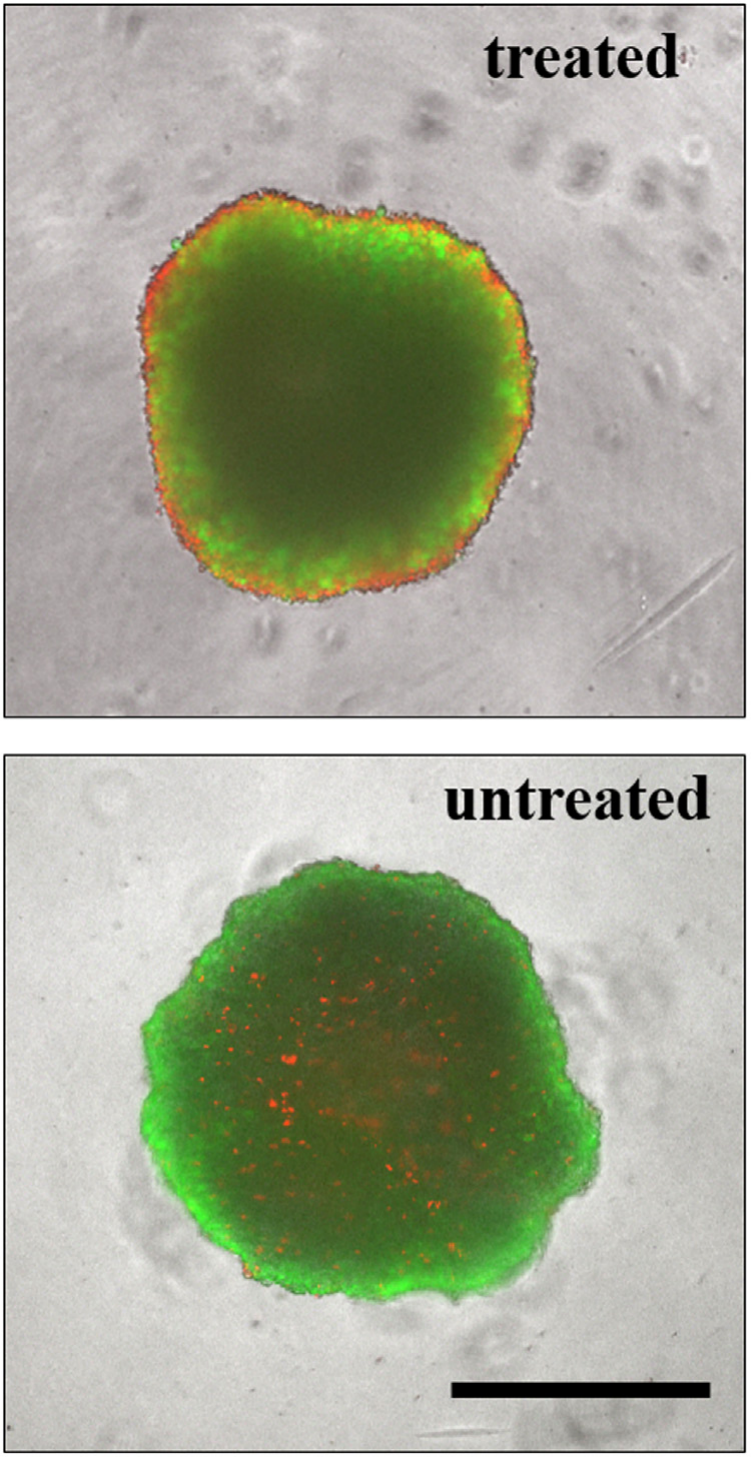
Fluorescence images of 100 μM Sorafenib-treated (top panel) and untreated (bottom panel) HepG2 spheroids after 6 days post-seeding using a calcein AM (green fluorescence) and ethidium bromide (red fluorescence) LIVE/DEAD assay. Live cells are monitored green and dead cells red. Scale bar, 500 μm.

Next, we generated uniformly-sized HepG2 spheroids in 96-well microtiter plates and exposed them to several doses of the anti-liver cancer drug sorafenib. An alamarBlue^®^ assay after 6 h and 24 h was performed to assess the influence of the incubation time on fluorescence intensity of HepG2 spheroids. Fig. 2 shows the measurement results comparing 2D monolayer with 3D spheroidal cultures of HepG2 cells. As shown in Fig. 2a, an extended incubation time of alamarBlue^®^-containing medium resulted in a significant improvement of assay reliability starting at a drug concentration of 50 μM (p < 0.05). It should also be noted that the assay precision increased tremendously by 12-fold extension of alamarBlue^®^ incubation time with an overall reduction of standard deviation range to 4–10%. In comparison, 2D monolayer cultures displayed similar comparable precision and reliability for any alamarBlue^®^ incubation time. Fig. 2b shows that optimization of the protocol for 3D spheroid cultures has a higher impact on the reliability and precision of the alamarBlue^®^ bioassay than longer exposure to drugs. For instance, no significant difference was observable for HepG2 spheroids exposed to sorafenib concentrations below 250 μM (p > 0.05). In comparison, 2D monolayer cultures showed a significant difference in drug-dose response between 24 h and longer exposure times already around 10 μM. Overall, these results indicate that the optimized protocol with an extension of incubation time to 24 h results in an improved and more reliable drug-dose response for 3D HepG2 spheroids. To confirm these viability results assayed by alamarBlue^®^, we compared the metabolic results with a LIVE/DEAD fluorescent viability kit. Fig. 3 shows morphological changes of HepG2 spheroids treated with 100 μM sorafenib for 24 h, analyzed with a LIVE/DEAD Viability/Cytotoxicity Kit for mammalian cells. The kit measures the cell viability based on the integrity of cell membranes similar to other dye-exclusion assays. The live cells are stained by Calcein AM, which emits green fluorescence light (517 nm) when excited by blue light (494 nm), while the dead cells are stained by Ethidium homodimer-1, which emit red fluorescence light (617 nm) when excited by green light (528 nm). Treated HepG2 spheroids displayed bright red fluorescent signal at the spheroid edges which corresponds to drug-induced apoptosis in comparison to untreated spheroids, which showed bright green fluorescent signal at the edges corresponding to living cell populations. Overall, this quantitative staining corresponds well with the optimized alamarBlue® assay protocol with a cell viability of 100 ± 9% for untreated and 42 ± 3% for treated HepG2 spheroids.

In summary, precise and reliable analysis of cell viability and proliferation for 3D cell cultures remains a challenging task. Here, we optimized the alamarBlue^®^ assay standard protocol to result in a more precise and reliable assay for drug efficacy testing in spheroid cultures using an optimized fluorescence-based metabolic assay.
